# Supplementary material for: Evolutionarily conserved regulation of immunity by the splicing factor RNP-6/PUF60
Source: eLife. 2020 Jun 15;9:e57591. doi: 10.7554/eLife.57591 (PMC7332298; doi:10.7554/eLife.57591)
Supplement: Supplementary file 1. [file elife-57591-supp1.docx]

| *C. elegans* Target | Assay | Forward sequence | Reverse sequence |
| --- | --- | --- | --- |
| *ilys-2* | qPCR | GTTGGATCGCTTTCTTGTGG | CGTCAGCACATCTCTTCCAG |
| *irg-1* | qPCR | TGATCTTGTTCCGTACCCATG | ATCCTCTCCAGTTTCGTTCATC |
| *spp-1* | qPCR | GGTGTTTTCTGTGATGTCTGC | ATAGTCCAGCAAAGAGTTCCG |
| *nlp-34* | qPCR | TCATCGCTTGCCTGTTGG | CATGGGCGGTAGTATGGG |
| *lys-3* | qPCR | CCAAGATATGATTAGAAGTGCGAAG | ACTAAACGTGTTCCAGCCTC |
| *irg-2* | qPCR | TGTTCGACGAGTTTTACTTCCG | CAATTGTGCCTTCAGTTTTCATG |
| *fmo-2* | qPCR | TGCCAAACAAGTCTACCTAGTC | TGTAGAGTGAGAAGAAACGCG |
| *nlp-30* | qPCR | TTCTCGCCTGCTTCATGG | GTCCATAACCTCCATATCCGC |
| *C18D11.6* | qPCR | AGTGGAAGCTAGTCAAACGC | GTGAAATCCCCAACCGAATG |
| *M01G12.9* | qPCR | CAGTATTGAAGGCGCATGTTC | TGATACTTTGTTAGCCCTCCATTG |
| *hpo-15* | qPCR | CGCTCTCCCATATTTCTCACTC | TCTGCGACCACTCAAATAACC |
| *Y43F8B.9* | qPCR | CTGCCAATGTGCTTTGAGTG | TCCAGTTTCCGCTTCACC |
| *fbxa-24* | qPCR | GAAGTACATTTCAAGGTTGCCG | GCATTTAATTACAAAGTTTGCATTATCG |
| *F47B8.4* | qPCR | TTGAGTATACATTGAATAAAATAGCATTCG | TCAGTTCTAATACGACGCCTTG |
| *F18G5.6* | qPCR | GAATTGAAGAGCTGAGAATGGC | TGGTGTTGGTATTGCTGAGTC |
| *F11E6.11* | qPCR | ACAGCTCTTTTGTCAATCTTTCAG | AGCAATCGGGCATTATACTTCC |
| *C07A4.3* | qPCR | GAAATGGTCGGATGAAATGGC | GAGATGGGAATTTTGAAGACGC |
| *M03B6.5* | qPCR | TGGAGCTTTCTGATTCATATCCTAG | ATTGAATTCCACTTTCTTCATCGTC |
| *lipl-2* | qPCR | TTACCAACTCAGAGTGCAGC | GCCATTTCATCCCAACTGAAC |
| *H11E01.2* | qPCR | CTGCGTACTGGGCTACATTC | GAGATAGGACAAAGTGGGAGTG |
| *cyp-37B1* | qPCR | AAGAATGTATCCGTCAGTGCC | AGATGAGAGGATTGCGTTGG |
| *cpz-2* | qPCR | GAAATCGGAAATGTGCTAGAGC | GTGTAGTTGGTAAGGGAGAAGC |
| *R10E8.8* | qPCR | AATCTTATAAAACAGGGATCGATTATACG | TCAGGTGCATTTCCGTATTCG |
| *rnp-6* | qPCR | CAGAAACAGCAACAGGAGAATC | GAAGCATATCTTCGCGGATTTC |
| *snb-1* | qPCR | GAATCATGAAGGTGAACGTGG | CCAATACTTGCGCTTCAGGG |
| *nhr-62* | qPCR | GAAAGTCAAGCAGCAGTTCG | ACTTGGCACAACGTGTAGG |
| *nhr-3* | qPCR | TTACGTCCAATTGCTGATTTGC | ATACTGTCGTGTTCTGGTGC |
| *cth-1* | qPCR | CATCTACACTTCATGCAACGTG | CTAGCGCGTTTTCAAAGTGAG |
| *fat-5* | qPCR | GGTGCTGATGTTCCAGAGG | CGTGTAGAAGGCGATGAAGG |
| *tos-1* | splicing | ATCTACGGATTCGAGTCGTCACCATC | GAAGAAATCTTCCAGTCCGAAGGG |
| *prg-2* | splicing | ATTGCTCGTCATCGGTGATA | CGATGATCCACAAACCATCA |
| *gyg-1* | splicing | TTCACACTGGCGAACACTTC | CTCATTGAGCGCTCTGTTCA |

| Human Target | Assay | Forward sequence | Reverse sequence |
| --- | --- | --- | --- |
| IL-6 | qPCR | CAACCTGAACCTTCCAAAGATG | ACCTCAAACTCCAAAAGACCAG |
| IL-8 | qPCR | TCCTGATTTCTGCAGCTCTG | GTCCACTCTCAATCACTCTCAG |
| IL-18 | qPCR | ATCGGCCTCTATTTGAAGATATGACT | CCTCTAGGCTGGCTATCTTTATACATACT |
| CXCL2 | qPCR | GGGCAGAAAGCTTGTCTCAA | GCTTCCTCCTTCCTTCTGGT |
| IL-1α | qPCR | CGCCAATGACTCAGAGGAAGA | AGGGCGTCATTCAGGATGAA |
| CCL5 | qPCR | TCTGCGCTCCTGCATCTG | GGGCAATGTAGGCAAAGCA |
| TGF-β1 | qPCR | CAATTCCTGGCGATACCTCAG | GCACAACTCCGGTGACATCAA |
| PUF60 | qPCR | GTTTCCCGAGTCAGAGCG | CGCAGAACCATCACTGTAGAC |
| GAPDH | qPCR | AATCCCATCACCATCTTCCA | TGGACTCCACGACGTACTCA |
| RPL13A | qPCR | CCTGGAGGAGAAGAGGAAAGAGA | TTGAGGACCTCTGTGTATTTGTCAA |

| Mouse target | Assay | Forward sequence | Reverse sequence |
| --- | --- | --- | --- |
| CCL-5 | qPCR | CCAATCTTGCAGTCGTGTTTGT | CATCTCCAAATAGTTGATGTATTCTTGAAC |
| IL-18 | qPCR | GCCATGTCAGAAGACTCTTGCGTC | GTACAGTGAAGTCGGCCAAAGTTGTC |
| CXCL2 | qPCR | GCGCTGTCAATGCCTGAAGA | TTTGACCGCCCTTGAGAGTG |
| IL-6 | qPCR | GAGGATACCACTCCCAACAGACC | AAGTGCATCATCGTTGTTCATACA |
| IL-1α | qPCR | TTGGTTAAATGACCTGCAACA | GAGCGCTCACGAACAGTTG |
| GAPDH | qPCR | AACGACCCCTTCATTGACCTC | ACTGTGCCGTTGAATTTGCC |
