# Supplementary material for: Evolutionarily conserved regulation of immunity by the splicing factor RNP-6/PUF60
Source: eLife. 2020 Jun 15;9:e57591. doi: 10.7554/eLife.57591 (PMC7332298; doi:10.7554/eLife.57591)
Supplement: Supplementary file 2. [file elife-57591-supp2.docx]

qPCR primer binding sites for *C. elegans* targets. For simplicity, only exons and variable UTRs are depicted.
