# Supplementary material for: Evolutionarily conserved regulation of immunity by the splicing factor RNP-6/PUF60
Source: eLife. 2020 Jun 15;9:e57591. doi: 10.7554/eLife.57591 (PMC7332298; doi:10.7554/eLife.57591)
Supplement: Supplementary file 5. [file elife-57591-supp5.docx]

| Antibody | SOURCE | IDENTIFIER |
| --- | --- | --- |
| Rat anti-HA | Roche | 3F10  RRID:AB_2314622 |
| Mouse anti-alpha tubulin | Sigma-Aldrich | DM1A RRID:AB_477593 |
| Rabbit anti-phospho-p38 MAPK (Thr180/Tyr182) | Cell Signaling Technology | #9211 RRID:AB_331641 |
| Mouse anti-beta Actin | Abcam | ab8224  RRID:AB_449644 |
| Rabbit anti-PUF60 | Thermo Fisher Scientific | PA5-21411  RRID:AB_11154782 |
| Rabbit anti-GAPDH | Cell Signaling Technology | #2118 RRID:AB_561053 |
| Mouse anti-FLAG | Sigma-Aldrich | M2  RRID:AB_262044 |
| Rabbit anti-PMK-1 | Read Pukkila-Worley lab, UMass Medical School | WormBase ID: [WBPaper00056073]::anti-PMK-1 |
| Anti-Mouse HRP | ThermoFisher | G-21040  RRID: AB_2536527 |
| Anti-Rabbit HRP | ThermoFisher | G-21234  RRID: AB_2536530 |
| Anti-Rat HRP | GE Healthcare | NA935  RRID: AB_772207 |
