# Supplementary material for: Evolutionarily conserved regulation of immunity by the splicing factor RNP-6/PUF60
Source: eLife. 2020 Jun 15;9:e57591. doi: 10.7554/eLife.57591 (PMC7332298; doi:10.7554/eLife.57591)
Supplement: Supplementary file 6. [file elife-57591-supp6.docx]

| **Median survival and p values for pathogen survival and ageing experiments. All experiments with *S. aureus*, *E. faecalis* and *P. aeruginosa* were performed at 25°C unless otherwise stated.** | | | |  |  |  |
| --- | --- | --- | --- | --- | --- | --- |
| Experiment | *p value* | Number of animals analyzed | Median survival (Hours) |  |  |  |
| *S. aureus* |  |  |  |  |  |  |
| Exp 1 N2 |  | 38 | 60 |  |  |  |
| vs. *rnp-6(dh1127)* | <0.0001 | 42 | 36 |  |  |  |
| Exp 2 N2 |  | 41 | 36 |  |  |  |
| vs. *rnp-6(dh1127)* | <0.0001 | 52 | 24 |  |  |  |
| Exp 3 N2 |  | 33 | 48 |  |  |  |
| vs. *rnp-6(dh1127)* | 0.0005 | 34 | 36 |  |  |  |
|  | | | |  |  |  |
| *E. faecalis* |  |  | Median survival (Days) |  |  |  |
| Exp 1 N2 |  | 49 | 6 |  |  |  |
| vs. *rnp-6(dh1127)* | <0.0001 | 57 | 4 |  |  |  |
| Exp 2 N2 |  | 41 | 5 |  |  |  |
| vs. *rnp-6(dh1127)* | 0.0431 | 40 | 4 |  |  |  |
| Exp 3 N2 |  | 48 | 5 |  |  |  |
| vs. *rnp-6(dh1127)* | <0.0001 | 49 | 4 |  |  |  |
|  | | | |  |  |  |
| *P. aeruginosa* |  |  | Median survival (Hours) |  |  |  |
| Exp 1 N2 |  | 33 | 60 |  |  |  |
| vs. *rnp-6(dh1127)* | <0.0001 | 33 | 48 |  |  |  |
| Exp 2 N2 |  | 28 | 48 |  |  |  |
| vs. *rnp-6(dh1127)* | 0.0008 | 21 | 36 |  |  |  |
| Exp 3 N2 |  | 43 | 48 |  |  |  |
| vs. *rnp-6(dh1127)* | <0.0001 | 46 | 36 |  |  |  |
|  | | | |  |  |  |
| *E. coli* 20°C |  |  | Median survival (Days) |  |  |  |
| Exp 1 N2 |  | 98 | 20 |  |  |  |
| vs. *rnp-6(dh1127)* | <0.0001 | 68 | 27 |  |  |  |
| Exp 2 N2 |  | 94 | 20 |  |  |  |
| vs. *rnp-6(dh1127)* | 0.0037 | 65 | 23 |  |  |  |
| Exp 3 N2 |  | 82 | 20 |  |  |  |
| vs. *rnp-6(dh1127)* | 0.0018 | 71 | 20 |  |  |  |
|  | | | |  |  |  |
| *S. aureus* |  |  | Median survival (Hours) |  |  |  |
| Exp 1 N2 Control-i |  | 51 | 48 |  |  |  |
| vs. N2 *rnp-6i* | <0.0001 | 46 | 60 |  |  |  |
| Exp 2 N2 Control-i |  | 41 | 36 |  |  |  |
| vs. N2 *rnp-6i* | <0.0001 | 45 | 60 |  |  |  |
| Exp 3 N2 Control-i |  | 51 | 36 |  |  |  |
| vs. N2 *rnp-6i* | <0.0001 | 44 | 60 |  |  |  |
|  | | | |  |  |  |
| *S. aureus* |  |  | Median survival (Hours) |  |  |  |
| Exp 1 N2 |  | 47 | 48 |  |  |  |
| vs. RNP-6 OE #1 | <0.0001 | 52 | 24 |  |  |  |
| vs. RNP-6 OE #2 | <0.0001 | 35 | 24 |  |  |  |
| vs. RNP-6 OE #3 | 0.0006 | 22 | 24 |  |  |  |
|  |  |  |  |  |  |  |
| Exp 2 N2 |  | 47 | 36 |  |  |  |
| vs. RNP-6 OE #1 | 0.0008 | 38 | 24 |  |  |  |
| vs. RNP-6 OE #2 | <0.0001 | 43 | 24 |  |  |  |
|  |  |  |  |  |  |  |
| Exp 3 N2 |  | 27 | 36 |  |  |  |
| vs. RNP-6 OE #1 | 0.0276 | 22 | 24 |  |  |  |
| vs. RNP-6 OE #2 | 0.0093 | 13 | 24 |  |  |  |
|  | | | |  |  |  |
| *E. coli* 25°C |  |  | Median survival (Days) |  |  |  |
| Exp 1 non-transgenic |  | 122 | 11 |  |  |  |
| vs. RNP-6 OE #1 | <0.0001 | 97 | 12 |  |  |  |
|  |  |  |  |  |  |  |
| Exp 2 non-transgenic |  | 117 | 8 |  |  |  |
| vs. RNP-6 OE #1 | <0.0001 | 102 | 11 |  |  |  |
|  |  |  |  |  |  |  |
| Exp 3 non-transgenic |  | 101 | 8 |  |  |  |
| vs. RNP-6 OE #1 | <0.0001 | 70 | 10 |  |  |  |
|  | | | |  |  |  |
| *S. aureus* |  |  | Median survival (Hours) |  |  |  |
| Exp 1 N2 Control-i |  | 23 | 36 |  |  |  |
| vs. N2 *rnp-6i* | 0.0003 | 38 | 72 |  |  |  |
| Exp 1 *pmk-1(km25)* Control-i |  | 48 | 24 |  |  |  |
| vs. *pmk-1 rnp-6i* | 0.1043 | 66 | 24 |  |  |  |
|  |  |  |  |  |  |  |
| Exp 2 N2 Control-i |  | 51 | 36 |  |  |  |
| vs. N2 *rnp-6i* | <0.0001 | 46 | 72 |  |  |  |
| Exp 2 *pmk-1(km25)* Control-i |  | 53 | 24 |  |  |  |
| vs. *pmk-1 rnp-6i* | 0.0567 | 50 | 24 |  |  |  |
|  |  |  |  |  |  |  |
| Exp 3 N2 Control-i |  | 48 | 36 |  |  |  |
| vs. N2 *rnp-6i* | 0.0005 | 43 | 60 |  |  |  |
| Exp 3 *pmk-1(km25)* Control-i |  | 50 | 24 |  |  |  |
| vs. *pmk-1 rnp-6i* | 0.2711 | 55 | 24 |  |  |  |
|  | | | |  |  |  |
| *S. aureus* |  |  | Median survival (Hours) |  |  |  |
| Exp 1 N2 Control-i |  | 43 | 36 |  |  |  |
| vs. N2 *rnp-6i* | <0.0001 | 43 | 72 |  |  |  |
| Exp 1 *tir-1(tm3036)* Control-i |  | 67 | 24 |  |  |  |
| vs. *tir-1* *rnp-6i* | 0.1074 | 70 | 24 |  |  |  |
|  |  |  |  |  |  |  |
| Exp 2 N2 Control-i |  | 39 | 36 |  |  |  |
| vs. N2 *rnp-6i* | 0.0016 | 54 | 36 |  |  |  |
| Exp 2 *tir-1(tm3036)* Control-i |  | 58 | 24 |  |  |  |
| vs. *tir-1 rnp-6i* | 0.9465 | 70 | 24 |  |  |  |
|  |  |  |  |  |  |  |
| Exp 3 *tir-1(tm3036)* Control-i |  | 41 | 24 |  |  |  |
| vs. *tir-1* *rnp-6i* | 0.4716 | 41 | 18 |  |  |  |
|  | | | |  |  |  |
| *S. aureus* |  |  | Median survival (Hours) |  |  |  |
| Exp 1 non-transgenic |  | 50 | 36 |  |  |  |
| vs. RNP-6 OE#1 | <0.0001 | 59 | 36 |  |  |  |
| Exp 1 *pmk-1(km25)* |  | 58 | 24 |  |  |  |
| vs. RNP-6 OE #1*;pmk-1* | 0.1692 | 64 | 24 |  |  |  |
|  |  |  |  |  |  |  |
| Exp 2 non-transgenic |  | 56 | 48 |  |  |  |
| vs. RNP-6 OE#1 | 0.0066 | 43 | 36 |  |  |  |
| Exp 2 *pmk-1(km25)* |  | 70 | 24 |  |  |  |
| vs. RNP-6 OE #1*;pmk-1* | 0.2007 | 69 | 18 |  |  |  |
|  |  |  |  |  |  |  |
| Exp 3 non-transgenic |  | 49 | 36 |  |  |  |
| vs. RNP-6 OE#1 | 0.0013 | 45 | 36 |  |  |  |
| Exp 3 *pmk-1(km25)* |  | 60 | 24 |  |  |  |
| vs. RNP-6 OE #1*;pmk-1* | 0.0532 | 60 | 18 |  |  |  |
|  | | | |  |  |  |
| *S. aureus* |  |  | Median survival (Hours) |  |  |  |
| Exp 1 N2 |  | 42 | 60 |  |  |  |
| vs. *rnp-6(dh1127)* | <0.0001 | 49 | 36 |  |  |  |
| Exp 1 *pmk-1(km25)* |  | 55 | 12 |  |  |  |
| vs. *rnp-6;pmk-1* | 0.1847 | 59 | 12 |  |  |  |
|  |  |  |  |  |  |  |
| Exp 2 N2 |  | 60 | 48 |  |  |  |
| vs. *rnp-6(dh1127)* | <0.0001 | 63 | 36 |  |  |  |
| Exp 2 *pmk-1(km25)* |  | 66 | 24 |  |  |  |
| vs. *rnp-6;pmk-1* | 0.1714 | 74 | 18 |  |  |  |
| Exp 3 N2 |  | 42 | 36 |  |  |  |
| vs. *rnp-6(dh1127)* | <0.0001 | 55 | 24 |  |  |  |
| Exp 3 *pmk-1(km25)* |  | 37 | 24 |  |  |  |
| vs. *rnp-6;pmk-1* | 0.0315 | 70 | 24 |  |  |  |
|  | | | |  |  |  |
| *P. aeruginosa* |  |  | Median survival (Hours) |  |  |  |
| Exp 1 N2 |  | 41 | 48 |  |  |  |
| vs. *rnp-6(dh1127)* | 0.0056 | 42 | 48 |  |  |  |
| Exp 1 *pmk-1(km25)* |  | 58 | 18 |  |  |  |
| vs. *rnp-6;pmk-1* | <0.0001 | 56 | 24 |  |  |  |
|  |  |  |  |  |  |  |
| Exp 2 N2 |  | 43 | 48 |  |  |  |
| vs. *rnp-6(dh1127)* | 0.0049 | 34 | 48 |  |  |  |
| Exp 2 *pmk-1(km25)* |  | 70 | 18 |  |  |  |
| vs. *rnp-6;pmk-1* | 0.003 | 69 | 24 |  |  |  |
|  |  |  |  |  |  |  |
| Exp 3 N2 |  | 37 | 60 |  |  |  |
| vs. *rnp-6(dh1127)* | <0.0001 | 42 | 48 |  |  |  |
| Exp 3 *pmk-1(km25)* |  | 63 | 24 |  |  |  |
| vs. *rnp-6;pmk-1* | 0.8109 | 66 | 36 |  |  |  |
|  | | | |  |  |  |
| *E. coli* 25°C |  |  | Median survival (Days) |  |  |  |
| Exp 1 non-transgenic |  | 107 | 10 |  |  |  |
| vs. SFA-1 OE | <0.0001 | 92 | 12 |  |  |  |
|  |  |  |  |  |  |  |
| Exp 2 non-transgenic |  | 108 | 8 |  |  |  |
| vs. SFA-1 OE | <0.0001 | 97 | 12 |  |  |  |
|  | | | |  |  |  |
| *S. aureus* |  |  | Median survival (Hours) |  |  |  |
| Exp 1 non-transgenic |  | 22 | 60 |  |  |  |
| vs. SFA-1 OE | 0.0165 | 18 | 36 |  |  |  |
|  |  |  |  |  |  |  |
| Exp 2 non-transgenic |  | 40 | 60 |  |  |  |
| vs. SFA-1 OE | <0.0001 | 34 | 36 |  |  |  |
|  |  |  |  |  |  |  |
| *S. aureus* |  |  | Median survival (Hours) |  |  |  |
| Exp 1 N2 Control-i |  | 43 | 48 |  |  |  |
| vs. N2 *sfa-1i* | 0.0048 | 50 | 60 |  |  |  |
|  |  |  |  |  |  |  |
| Exp 2 N2 Control-i |  | 38 | 36 |  |  |  |
| vs. N2 *sfa-1i* | <0.0001 | 43 | 84 |  |  |  |
|  | | | |  |  |  |
| *S. aureus* |  |  | Median survival (Hours) |  |  |  |
| Exp 1 N2 Control-i |  | 62 | 48 |  |  |  |
| vs. *rnp-6(dh1127)* Control-i | 0.0002 | 59 | 36 |  |  |  |
| Exp 1 N2 *sfa-1i* |  | 54 | 48 |  |  |  |
| vs. *rnp-6(dh1127) sfa-1i* | 0.0757 | 44 | 60 |  |  |  |
|  |  |  |  |  |  |  |
| Exp 2 N2 Control-i |  | 43 | 48 |  |  |  |
| vs. *rnp-6(dh1127)* Control-i | 0.0339 | 55 | 36 |  |  |  |
| Exp 2 N2 *sfa-1i* |  | 50 | 60 |  |  |  |
| vs. *rnp-6(dh1127) sfa-1i* | 0.0867 | 37 | 72 |  |  |  |
|  |  |  |  |  |  |  |
| Exp 3 N2 Control-i |  | 36 | 36 |  |  |  |
| vs. *rnp-6(dh1127)* Control-i | 0.0026 | 41 | 36 |  |  |  |
| Exp 3 N2 *sfa-1i* |  | 42 | 48 |  |  |  |
| vs. *rnp-6(dh1127) sfa-1i* | 0.443 | 46 | 48 |  |  |  |
|  | | | |  |  |  |
| *E. coli* 20°C |  |  | Median survival (Days) |  |  |  |
| Exp 1 non-transgenic |  | 85 | 13 |  |  |  |
| vs. SFA-1 OE | <0.0001 | 81 | 20 |  |  |  |
| Exp 1 *rnp-6(dh1127)* |  | 79 | 18 |  |  |  |
| vs. *rnp-6*; SFA-1 OE | 0.0147 | 87 | 16 |  |  |  |
|  |  |  |  |  |  |  |
| Exp 2 non-transgenic |  | 98 | 11 |  |  |  |
| vs. SFA-1 OE | <0.0001 | 73 | 18 |  |  |  |
| Exp 2 *rnp-6(dh1127)* |  | 33 | 22 |  |  |  |
| vs. *rnp-6*; SFA-1 OE | <0.0001 | 73 | 18 |  |  |  |
|  | | | |  |  |  |
| *E. coli* 20°C |  |  | Median survival (Days) |  |  |  |
| Exp 1 N2 Control-i |  | 90 | 20 |  |  |  |
| vs. *rnp-6(dh1127)* Control-i | <0.0001 | 72 | 22 |  |  |  |
| Exp 1 N2 *sfa-1i* |  | 100 | 20 |  |  |  |
| vs. *rnp-6(dh1127) sfa-1i* | <0.0001 | 129 | 15 |  |  |  |
|  |  |  |  |  |  |  |
| Exp 2 N2 Control-i |  | 95 | 21 |  |  |  |
| vs. *rnp-6(dh1127)* Control-i | 0.0001 | 117 | 25 |  |  |  |
| Exp 2 N2 *sfa-1i* |  | 85 | 21 |  |  |  |
| vs. *rnp-6(dh1127) sfa-1i* | <0.0001 | 106 | 15 |  |  |  |
|  |  |  |  |  |  |  |
| Exp 3 N2 *sfa-1i* |  | 47 | 19 |  |  |  |
| vs. *rnp-6(dh1127) sfa-1i* | <0.0001 | 49 | 14 |  |  |  |
|  | | | |  |  |  |
| *S. aureus* |  |  | Median survival (Hours) |  |  |  |
| Exp 1 non-transgenic |  | 64 | 48 |  |  |  |
| vs. SFA-1 OE#1 | <0.0001 | 62 | 36 |  |  |  |
| Exp 1 *pmk-1(km25)* |  | 71 | 18 |  |  |  |
| vs. SFA-1 OE #1*;pmk-1* | 0.2206 | 68 | 24 |  |  |  |
|  |  |  |  |  |  |  |
| Exp 2 non-transgenic |  | 57 | 60 |  |  |  |
| vs. SFA-1 OE#1 | <0.0001 | 53 | 36 |  |  |  |
| Exp 2 *pmk-1(km25)* |  | 70 | 24 |  |  |  |
| vs. SFA-1 OE #1*;pmk-1* | 0.0001 | 54 | 24 |  |  |  |
|  |  |  |  |  |  |  |
| Exp 3 non-transgenic |  | 56 | 48 |  |  |  |
| vs. SFA-1 OE#1 | 0.0013 | 53 | 36 |  |  |  |
| Exp 3 *pmk-1(km25)* |  | 64 | 24 |  |  |  |
| vs. SFA-1 OE #1*;pmk-1* | 0.1344 | 57 | 24 |  |  |  |
|  | | | |  |  |  |
| *S. aureus* |  |  | Median survival (Hours) |  |  |  |
| Exp 1 N2 Control-i |  | 40 | 36 |  |  |  |
| vs. N2 *sfa-1i* | <0.0001 | 50 | 60 |  |  |  |
| Exp 1 *pmk-1(km25)* Control-i |  | 56 | 24 |  |  |  |
| vs. *pmk-1 sfa-1i* | <0.0001 | 51 | 36 |  |  |  |
|  |  |  |  |  |  |  |
| Exp 2 N2 Control-i |  | 37 | 36 |  |  |  |
| vs. N2 *sfa-1i* | 0.0011 | 44 | 48 |  |  |  |
| Exp 2 *pmk-1(km25)* Control-i |  | 55 | 24 |  |  |  |
| vs. *pmk-1 sfa-1i* | <0.0001 | 58 | 36 |  |  |  |
|  |  |  |  |  |  |  |
| Exp 3 N2 Control-i |  | 50 | 36 |  |  |  |
| vs. N2 *sfa-1i* | <0.0001 | 48 | 48 |  |  |  |
| Exp 3 *pmk-1(km25)* Control-i |  | 51 | 18 |  |  |  |
| vs. *pmk-1 sfa-1i* | <0.0001 | 59 | 24 |  |  |  |
|  | | | |  |  |  |
| *E. coli* 25°C |  |  | Median survival (Days) |  |  |  |
| Exp 1 N2 |  | 109 | 14 |  |  |  |
| vs. *rnp-6(dh1127)* | 0.3102 | 92 | 14 |  |  |  |
| Exp 2 N2 |  | 121 | 11 |  |  |  |
| vs. *rnp-6(dh1127)* | 0.0734 | 124 | 11 |  |  |  |
|  | | | |  |  |  |
| *S. aureus* 20°C |  |  | Median survival (Hours) |  |  |  |
| Exp 1 N2 |  | 32 | 60 |  |  |  |
| vs. *rnp-6(dh1127)* | <0.0001 | 26 | 36 |  |  |  |
| Exp 2 N2 |  | 51 | 60 |  |  |  |
| vs. *rnp-6(dh1127)* | 0.0079 | 46 | 48 |  |  |  |
| Exp 3 N2 |  | 30 | 84 |  |  |  |
| vs. *rnp-6(dh1127)* | 0.0046 | 18 | 60 |  |  |  |
|  |  |  |  |  |  |  |
| *S. aureus* |  |  | Median survival (Hours) |  |  |  |
| Exp 1 N2 |  | 32 | 60 |  |  |  |
| vs. *rnp-6(dh1125)* | 0.0008 | 13 | 24 |  |  |  |
| Exp 2 N2 |  | 42 | 36 |  |  |  |
| vs. *rnp-6(dh1125)* | 0.0076 | 43 | 24 |  |  |  |
|  | | | |  |  |  |
| *E. faecalis* |  |  | Median survival (Days) |  |  |  |
| Exp 1 N2 |  | 44 | 5 |  |  |  |
| vs. *rnp-6(dh1125)* | 0.0049 | 38 | 4 |  |  |  |
| Exp 2 N2 |  | 49 | 6 |  |  |  |
| vs. *rnp-6(dh1125)* | <0.0001 | 58 | 3 |  |  |  |
| Exp 3 N2 |  | 41 | 5 |  |  |  |
| vs. *rnp-6(dh1125)* | 0.0293 | 46 | 4 |  |  |  |
|  | | | |  |  |  |
| *P. aeruginosa* |  |  | Median survival (Hours) |  |  |  |
| Exp 1 N2 |  | 43 | 48 |  |  |  |
| vs. *rnp-6(dh1125)* | <0.0001 | 45 | 36 |  |  |  |
| Exp 2 N2 |  | 33 | 60 |  |  |  |
| vs. *rnp-6(dh1125)* | 0.0022 | 16 | 48 |  |  |  |
|  | | | |  |  |  |
| *S. aureus* full lawn  FUDR sterilized animals |  |  | Median survival (Hours) |  |  |  |
| Exp 1 N2 |  | 47 | 60 |  |  |  |
| vs. *rnp-6(dh1127)* | <0.0001 | 50 | 48 |  |  |  |
| Exp 2 N2 |  | 38 | 180 |  |  |  |
| vs. *rnp-6(dh1127)* | <0.0001 | 48 | 84 |  |  |  |
| Exp 3 N2 |  | 43 | 204 |  |  |  |
| vs. *rnp-6(dh1127)* | <0.0001 | 48 | 108 |  |  |  |
|  | | | |  |  |  |
| *S. aureus* full lawn |  |  | Median survival (Hours) |  |  |  |
| Exp 1 N2 Control-i |  | 47 | 48 |  |  |  |
| vs. N2 *rnp-6i* | <0.0001 | 53 | 60 |  |  |  |
| Exp 2 N2 Control-i |  | 56 | 36 |  |  |  |
| vs. N2 *rnp-6i* | 0.0204 | 50 | 48 |  |  |  |
| Exp 3 N2 Control-i |  | 36 | 72 |  |  |  |
| vs. N2 *rnp-6i* | 0.0329 | 43 | 84 |  |  |  |
|  | | | |  |  |  |
| *E. faecalis* |  |  | Median survival (Days) |  |  |  |
| Exp 1 non-transgenic |  | 61 | 6 |  |  |  |
| vs. RNP-6 OE #1 | <0.0001 | 48 | 4 |  |  |  |
| Exp 2 non-transgenic |  | 61 | 5 |  |  |  |
| vs. RNP-6 OE #1 | <0.0001 | 56 | 3 |  |  |  |
| Exp 3 non-transgenic |  | 61 | 5 |  |  |  |
| vs. RNP-6 OE #1 | <0.0001 | 59 | 3 |  |  |  |
|  | | | |  |  |  |
| *E. faecalis* |  |  | Median survival (Days) |  |  |  |
| Exp 1 non-transgenic |  | 50 | 6 |  |  |  |
| vs. RNP-6 OE #2 | <0.0001 | 53 | 2 |  |  |  |
| Exp 2 non-transgenic |  | 53 | 5 |  |  |  |
| vs. RNP-6 OE #2 | <0.0001 | 61 | 2 |  |  |  |
| Exp 3 non-transgenic |  | 61 | 5 |  |  |  |
| vs. RNP-6 OE #2 | <0.0001 | 63 | 2 |  |  |  |
|  | | | |  |  |  |
| *S. aureus* full lawn  FUDR sterilized animals |  |  | Median survival (Hours) |  |  |  |
| Exp 1 non-transgenic |  | 67 | 84 |  |  |  |
| vs. RNP-6 OE #1 | <0.0001 | 57 | 72 |  |  |  |
| Exp 2 non-transgenic |  | 48 | 48 |  |  |  |
| vs. RNP-6 OE #1 | 0.0052 | 45 | 36 |  |  |  |
| Exp 3 non-transgenic |  | 59 | 60 |  |  |  |
| vs. RNP-6 OE #1 | 0.0539 | 54 | 48 |  |  |  |
|  |  |  |  |  |  |  |
| *S. aureus* |  |  | Median survival (Hours) |  |  |  |
| Exp 1 *daf-16(mu86)* Control-i |  | 39 | 36 |  |  |  |
| vs. *daf-16 rnp-6i* | <0.0001 | 30 | 84 |  |  |  |
| Exp 2 *daf-16(mu86)* Control-i |  | 31 | 36 |  |  |  |
| vs. *daf-16 rnp-6i* | 0.0028 | 23 | 72 |  |  |  |
| Exp 3 *daf-16(mu86)* Control-i |  | 24 | 36 |  |  |  |
| vs. *daf-16 rnp-6i* | <0.0001 | 27 | 84 |  |  |  |
|  |  |  |  |  |  |  |
| *S. aureus* |  |  | Median survival (Hours) |  |  |  |
| Exp 1 *hlh-30(tm1978)* Control-i |  | 40 | 24 |  |  |  |
| vs. *hlh-30 rnp-6i* | <0.0001 | 40 | 36 |  |  |  |
| Exp 2 *hlh-30(tm1978)* Control-i |  | 53 | 24 |  |  |  |
| vs. *hlh-30 rnp-6i* | <0.0001 | 47 | 36 |  |  |  |
| Exp 3 *hlh-30(tm1978)* Control-i |  | 63 | 24 |  |  |  |
| vs. *hlh-30 rnp-6i* | <0.0001 | 66 | 36 |  |  |  |
|  |  |  |  |  |  |  |
| *S. aureus* |  |  | Median survival (Hours) |  |  |  |
| Exp 1 N2 Control-i |  | 44 | 36 |  |  |  |
| vs. N2 *repo-1i* | 0.0005 | 54 | 48 |  |  |  |
| vs. N2 *hrpu-1i* | 0.0691 | 37 | 36 |  |  |  |
| Exp 1 *rnp-6(dh1127)* Control-i |  | 59 | 24 |  |  |  |
| vs. *rnp-6* *repo-1i* | 0.0001 | 58 | 36 |  |  |  |
| vs. *rnp-6* *hrpu-1i* | 0.0081 | 38 | 36 |  |  |  |
|  |  |  |  |  |  |  |
| Exp 2 N2 Control-i |  | 36 | 36 |  |  |  |
| vs. N2 *repo-1i* | 0.0757 | 36 | 48 |  |  |  |
| vs. N2 *hrpu-1i* | 0.0002 | 35 | 36 |  |  |  |
| Exp 2 *rnp-6(dh1127)* Control-i |  | 41 | 36 |  |  |  |
| vs. *rnp-6* *repo-1i* | 0.4407 | 57 | 36 |  |  |  |
| vs. *rnp-6* *hrpu-1i* | 0.1179 | 59 | 36 |  |  |  |
|  |  |  |  |  |  |  |
| Exp 3 N2 Control-i |  | 54 | 36 |  |  |  |
| vs. N2 *repo-1i* | 0.0048 | 57 | 48 |  |  |  |
| vs. N2 *hrpu-1i* | <0.0001 | 43 | 36 |  |  |  |
| Exp 3 *rnp-6(dh1127)* Control-i |  | 63 | 36 |  |  |  |
| vs. *rnp-6* *repo-1i* | 0.6069 | 58 | 36 |  |  |  |
| vs. *rnp-6* *hrpu-1i* | <0.0001 | 45 | 24 |  |  |  |
